# Depth of response may predict clinical outcome in patients with recurrent/metastatic head and neck cancer treated with pembrolizumab-containing regimens

**DOI:** 10.3389/fonc.2023.1230731

**Published:** 2023-08-16

**Authors:** Ken Saijo, Hiroo Imai, Kota Ouchi, Keiju Sasaki, Yuya Yoshida, Yoshifumi Kawamura, Sakura Taniguchi, Yuki Kasahara, Keigo Komine, Hidekazu Shirota, Masanobu Takahashi, Chikashi Ishioka

**Affiliations:** ^1^ Department of Medical Oncology, Tohoku University Hospital, Sendai, Japan; ^2^ Department of Clinical Oncology, Tohoku University Graduate school of Medicine, Sendai, Japan

**Keywords:** immune check point inhibitor, pembrolizumab, head and neck cancer, depth of response, predictive factor

## Abstract

**Background:**

Pembrolizumab-containing regimens are standards of care for recurrent and metastatic head and neck squamous cell carcinoma (R/M HNSCC). The depth of response (DpR) predicts the survival of patients with several types of solid cancers; however, its association with the survival outcomes of patients with R/M HNSCC treated with pembrolizumab-containing regimens remains unclear.

**Methods:**

This study included 66 patients with R/M HNSCC who received a pemblolizumab-containing regimen as a first-line therapy at Tohoku University Hospital, Sendai, Japan. The patients’ characteristics, combined positive score, baseline tumor size, tumor response, DpR, overall survival (OS), progression-free survival (PFS), PFS2, and adverse events were reviewed. The associations between DpR and survival outcomes were analyzed.

**Results:**

The 1 year-OS and 1 year-PFS rates of pembrolizumab-containing regimens were 69.4% and 24.4%, respectively. The response rate was 28.8%. The mean and median values of tumor change from baseline were 5.1% and −9.0%. In the correlation analysis, a significant negative correlation was observed between tumor change rate from baseline and survival outcomes (OS: r= −0.41, p=0.0017; PFS: r=−0.49, p<0.001). In the multivariate analysis, DpR with tumor change of ≤−45 was associated with better OS and PFS.

**Conclusion:**

DpR induced by pembrolizumab-containing regimens may be a predictive factor for OS and PFS in patients with R/M HNSCC.

## 1 Introduction

Immune checkpoint inhibitors (ICIs) play a crucial role in the treatment of many types of cancer, including recurrent and metastatic head and neck squamous cell carcinoma (R/M HNSCC) (1). Pembrolizumab is a monoclonal antibody that targets programmed cell death protein 1 (PD-1), expressed in immune cells. In the tumor immune microenvironment, PD-1 interacts with programmed death ligand-1 (PD-L1), expressed on cancer cells, enabling them to evade the immune system (2). Pembrolizumab disrupts the interaction between PD-1 and PD-L1, leading to an antitumor effect through reactivation of the immune system. Pembrolizumab, with or without chemotherapy, is the standard first-line treatment for R/M HNSCC based on the results of the KEYNOTE-048 study (3). Recently, it has been reported pembrolizumab-containing regimens provided a survival benefit of over 4 years in an updated analysis of the KEYNOTE-048 study (4). A subgroup analysis performed in the KEYNOTE-048 study showed that the PD-L1 combined positive score (CPS) was useful in predicting the efficacy of pembrolizumab-containing regimens (5). However, the exploration of additional predictive biomarkers is necessary for appropriate use of pembrolizumab-containing regimens and establishing overall treatment strategies for R/M HNSCC.

The depth of response (DpR) represents the percentage of tumor shrinkage observed at the nadir compared with the baseline. DpR was initially reported as a surrogate marker of survival in metastatic colorectal cancer (6). Since then, the correlation between DpR and survival benefits has been investigated in other types of cancers, such as melanoma, lung, gastric, breast, and pancreatic cancers (7, 8). However, in R/M HNSCC, the association of DpR and pembrolizumab regimens with survival outcomes remains unclear.

Hence, we retrospectively reviewed the clinical records of patients with R/M HNSCC who received a pembrolizumab-containing regimen in our institute and analyzed the association between DpR and survival outcomes. This study is the first to document the association of DpR upon administration of a pembrolizumab-containing regimen with the clinical survival of patients with R/M HNSCC.

## 2 Materials and methods

### 2.1 Patients and study design

We retrospectively identified 66 patients with R/M HNSCC who received pembrolizumab, with or without chemotherapy, as first-line therapy at Tohoku University Hospital, Sendai, Japan. Data on age, sex, Eastern Cooperative Oncology Group (ECOG) performance status (PS), primary site, p16 status in oropharyngeal cancer, metastatic sites, CPS in tumor tissue, overall survival (OS), progression-free survival (PFS), progression-free survival from first line on second-line therapy (PFS2), target lesions, response, and adverse events (AEs) were collected. The target lesions and treatment response were evaluated according to the Response Evaluation Criteria in Solid Tumors (RECIST) version 1.1. The target lesions were set to maximum of five total, including maximum of two per organ. Lymph nodes with a short diameter of less than 10 mm on computed tomography were considered normal. Baseline tumor size was quantified as the sum of the diameters of target lesions (9). Based on the target lesions, tumor change rate was calculated. DpR was defined as the best tumor change rate observed at any time point during the first-line therapy. The AEs were evaluated and graded according to the National Cancer Institute Common Terminology Criteria for Adverse Events version 5.0. PD-L1 expression was determined immunohistochemically using surgically resected or biopsied formalin-fixed paraffin-embedded tissues. CPS was calculated as the number of PD-L1-stained cells among tumor cells, lymphocytes, and macrophages divided by the total number of tumor cells and multiplied by 100 (10). This study was approved by the Ethics Committee of the Faculty of Medicine of the Tohoku University School of Medicine (approval no. 2021-1-351) and conducted in accordance with the ethical standards described in the Declaration of Helsinki. Patient consent for this study was obtained using the opt-out method.

### 2.2 Treatment regimen

In the pembrolizumab with chemotherapy and pembrolizumab alone regimens, pembrolizumab (200 mg) was administered once every 3 weeks. The chemotherapy regimen combined with pembrolizumab consisted of 5-fluorouracil (continuous infusion of 1,000 mg/m^2^ per day for four days) and either cisplatin (100 mg/m^2^ on day 1) or carboplatin (area under the curve of 5 mg/m^2^ on day 1) every 3 weeks. In the pembrolizumab with chemotherapy regimen, sequential maintenance therapy with pembrolizumab (200 mg) every 3 weeks was planned after six courses of pembrolizumab plus a chemo-combination phase.

### 2.3 Statistical analyses

OS and PFS were estimated using the Kaplan-Meier method and compared using log-rank tests, with p<0.05 considered statistically significant. The correlations between DpR and survival time and between DpR and baseline tumor size were estimated using the Pearson correlation coefficient. In the contingency table, statistical significance was evaluated using Fisher’s exact test. All statistical analyses were performed using the JMP Pro15 software (SAS Institute Inc., Cary, NC, USA).

## 3 Results

### 3.1 Patients’ clinical characteristics

The patients’ characteristics are presented in **Table 1**. The median patient age was 69 years (range: 21–88). The patients were predominantly male (n=52, 78.8%). The majority of the patients had an ECOG PS of 0 or 1 (n=57, 86.4%). The median baseline tumor size was 31mm. The CPS was ≥20 in 34 patients (51.5%), ≥1 in 55 (83.3%), <1 in 5 (7.6%); meanwhile, the CPS was not evaluated in 6 patients (9.1%). The primary sites were the oral cavity in 26 patients (39.4%), hypopharynx in 22 (33.3%), oropharynx in 7 (10.6%), larynx in 3 (4.5%), nasopharynx in 3 (4.5%), maxillary sinus in 1 (1.5%), nasal cavity in 1 (1.5%), and unknown in 3 (4.5%). Among 7 patients with oropharyngeal cancer, four had a p16-positive status. The metastatic sites were the lungs in 24 patients (36.4%), lymph nodes in 35 (53.0%), liver in 3 (4.5%), and bone in 10 (15.2%); local recurrence was reported in 17 patients (25.8%). With regard to the treatment regimen, 40 patients received pembrolizumab with chemotherapy, while 26 received pembrolizumab alone. Of the 40 patients who received pembrolizumab in combination with chemotherapy, 38 (95.0%) received carboplatin-based chemotherapy. Only two patients received cisplatin-based chemotherapy. All patients with a CPS of <1 received pembrolizumab with chemotherapy. At the time of analysis, subsequent anticancer therapy was received by 33 (50.0%) patients. The most common subsequent regimen was cetuximab plus paclitaxel received by 23 (34.8%).

**Table 1:** Patient characteristics.

pembrolizumab-containing regimen	
	n=66
Age	
median (range)	69 (21-88)
Sex, n (%)	
male	55 (83.3)
female	11 (16.6)
ECOG PS, n (%)	
0	19 (28.8)
1	38 (57.6)
2	8 (12.1)
3	1 (1.5)
Baseline tumor size, mm	
median (range)	31 (9-144)
CPS, n (%)	
≥20	34 (51.5)
≥1	55 (83.3)
<1	5 (7.6)
NE	6 (9.1)
Primary site, n (%)	
oral cavity	26 (39.4)
hypopharynx	22 (33.3)
oropharynx	7 (10.6)
P16 positive	4 (6.1)
larynx	3 (4.5)
nasopharynx	3 (4.5)
maxillary sinus	1 (1.5)
nasal cavity	1 (1.5)
unknown primary	3 (4.5)
metastaic site n, (%)	
lung	24 (36.4)
lymph node	35 (53)
liver	3 (4.5)
bone	10 (15.2)
locoregional	17 (25.8)
regimen n, (%)	
pembrolizumab alone	26 (39.4)
pembrolziumab with chemotherapy	40 (60.6)
5FU + cisplatin	2 (3.1)
5FU + carboplatin	38 (57.5)
subsequent therapy n, (%)	47 (71.2)
anticancer therapy	33 (50.0)
cetuximab + paclitaxel	23 (34.8)
paclitaxel	4 (6.1)
docetaxel	2 (3.0)
cetuximab + 5FU + cisplatin	2 (3.0)
5FU + cisplatin	1 (1.5)
clinical trial	1 (1.5)
best supportive care	14 (21.2)

OS, overall survival; PFS, progression free survival; HR, hazard ratio; CI, confidence interval; PS, performance status; CPS, combined positive score; NE, not evaluated.

### 3.2 Treatment efficacies of pembrolizumab-containing regimens

**Figure 1:**
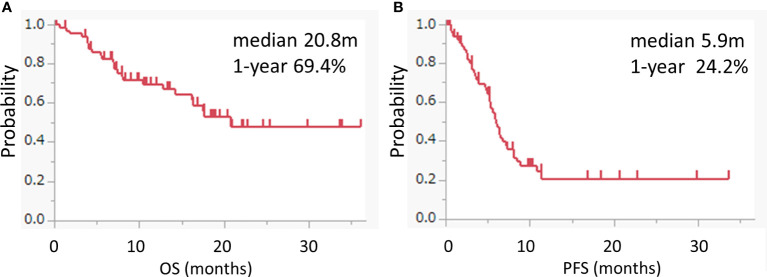
Kaplan–Meier survival curves for OS and PFS. **(A, B)** Kaplan–Meier curves for the OS **(A)** and PFS **(B)** of the pembrolizumab-containing regimen. Pembrolizumab-containing regimens included both pembrolizumab with chemotherapy and pembrolizumab alone.

**Figure 2:**
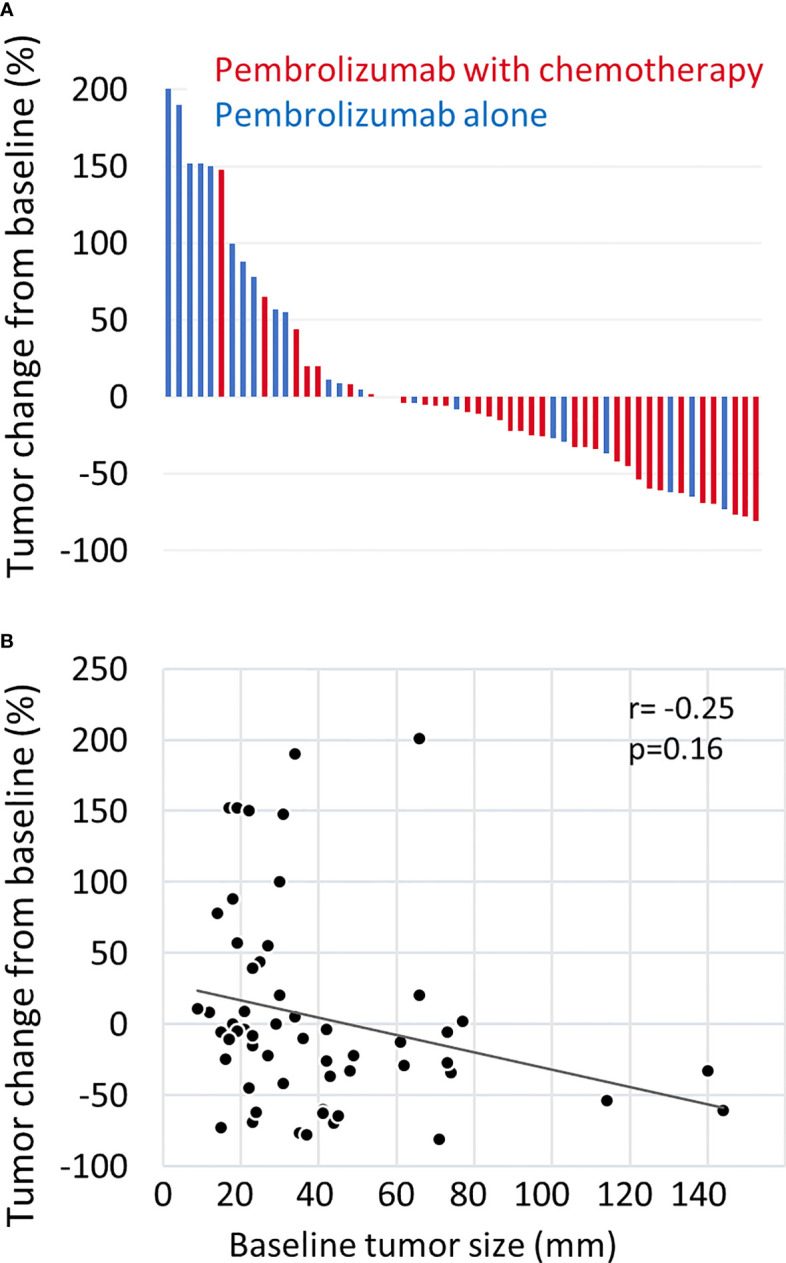
Waterfall plot of tumor measurements. **(A)** The maximal change rate in the tumor from the baseline after treatment with pembrolizumab plus chemotherapy (red) or pembrolizumab alone (blue) is shown. Tumor changes were evaluated according to the RECIST version 1.1. Patients without tumor response evaluation were excluded. **(B)** The correlation between maximal tumor change from baseline and baseline tumor size was estimated by the Pearson correlation coefficient. Baseline tumor size was quantified as the sum of the length diameters of RECIST target lesions.

The median OS was 20.8 months (95% CI: 14.2-not applicable), and the 1 year-OS rate was 69.4% (**Figure 1A**). The median PFS was 5.9 months (95% CI: 5.2-7.2), and the 1 year-PFS rate was 24.4% (**Figure 1B**). The OS and PFS of pembrolizumab with chemotherapy and pembrolizumab alone are shown in **Supplementary Figure 1**. The median OS and PFS of patients who received pembrolizumab with chemotherapy were 20.8 (95% CI: 14.2-not applicable) and 6.3 (95% CI: 5.3-8.1) months, respectively. The median OS was not reached, while the median PFS was 5.2 (95% CI: 2.5-not applicable) months in patients who received pembrolizumab alone. No complete responses were observed. Partial responses were observed in 19 patients (28.8%), followed by stable disease in 25 patients (37.9%), and progressive disease in 13 patients (19.7%). The response rate was 28.8%. The disease control rate was 66.7%. The maximal tumor changes from the baseline are shown in a waterfall plot in **Figure 2A**. The mean tumor change rates for the total pembrolizumab-containing regimen, pembrolizumab with chemotherapy, and pembrolizumab alone were 5.1%, −18.8%, and 44.9%, respectively. The median time to response was 2.0 months (95% CI: 1.8-2.5). The median time to best response for the measurement of DpR was 2.8 (95% CI: 1.9-4.7) months. The median duration of response was 5.5 months (95% CI: 3.2-9.5) months. A correlation analysis was performed to verify the correlation between tumor change rates and baseline tumor size. As a result, no significant correlation was observed (p=0.160) (**Figure 2B**).

### 3.3 Correlation between DpR and survival outcome

**Figure 3:**
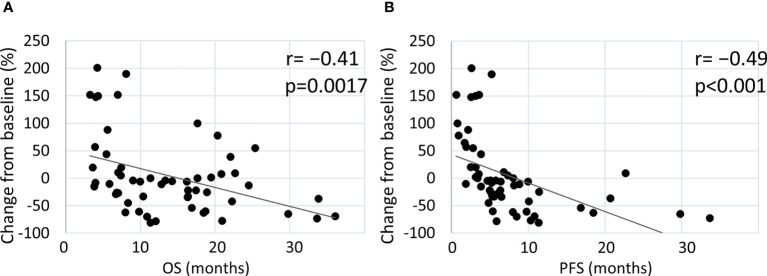
Estimated correlation between maximal tumor change from the baseline and clinical outcome. Correlation of tumor changes following treatment with the pembrolizumab-containing regimen with OS **(A)** and PFS **(B)**.

**Figure 3** shows the correlation between tumor changes from the baseline and survival outcomes. In all patients treated with the pembrolizumab-containing regimen, a significant negative correlation was found between tumor changes and survival outcomes (OS: r=−0.41, p=0.0017; PFS: r=−0.49, p<0.001) (**Figures 3A, B**). In patients who received pembrolizumab with chemotherapy, a minor negative correlation was observed between tumor changes and OS (r=−0.35, p=0.037), while a negative correlation was observed between tumor changes and PFS (r=−0.59, p<0.001) (**Supplementary Figures 2A, B**). In patients who received pembrolizumab alone, a negative correlation was observed between OS (r=−0.47, p=0.030) and PFS (r=−0.62, p=0.030) (**Supplementary Figures 2C, D**). Thus, a deeper response to the pembrolizumab regimen was significantly associated with better survival outcomes.

### 3.4 Univariate and multivariate analyses

**Figure 4:**
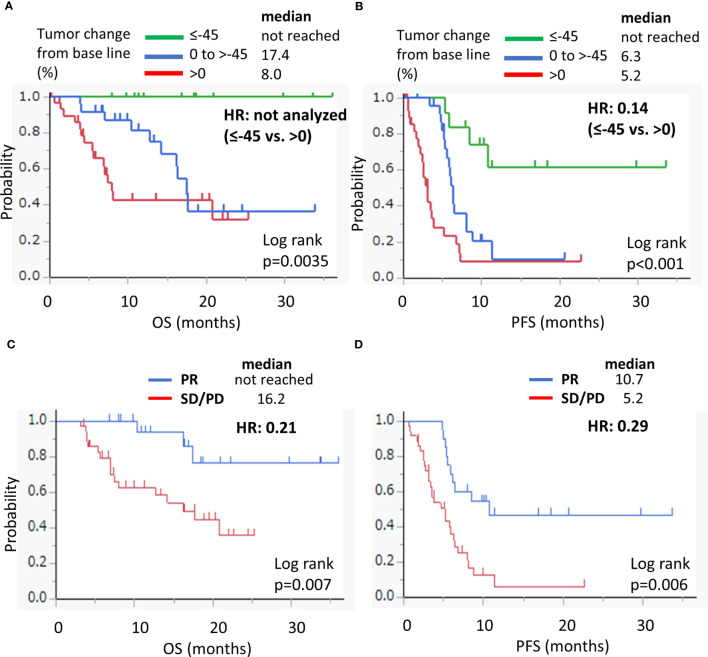
Kaplan–Meier survival curves for OS **(A)** and PFS **(B)** according to the depth of response on the treatment with pembrolizumab-containing regimen. Kaplan–Meier survival curves for OS **(C)** and PFS **(D)** according to RECIST-based criteria (PR vs. SD/PD). OS, overall survival; PR, partial response; SD, stable disease; PD, progressive disease; HR, hazard ratio.

Univariate and multivariate analyses of the OS and PFS were performed (**Table 2**). In the univariate analysis of OS, an ECOG PS score of 0, the presence of distant metastasis, and a tumor change rate of −45% or less were significantly associated with better OS. The hazard ratio values gradually decreased as the tumor shrinkage rate increased. Baseline tumor sizes with a continuous value and with a cutoff of 31 mm (median value) were not associated with OS. CPS with cut-off values of 1 and 20, primary sites, and occurrence of severe AEs were also not associated with OS. Locoregional recurrence was associated with a worse OS. In the multivariate analysis including three explanatory variables such as PS, baseline tumor size, and DpR, only DpR with a tumor change of −45% or less was a significant predictor of better OS. As for PFS, a tumor change of 0% to −15% and a change of −45% or less were associated with better PFS in the univariate analysis. Locoregional recurrence was associated with a worse PFS. In the multivariate analysis, the tumor change of −45% or less was an independent predictor of better PFS. Kaplan–Meier analysis revealed that the OS and PFS were significantly longer in patients with a DpR of tumor change of −45% or less (**Figures 4A, B**). Moreover, no events were observed in the OS analysis of these patients (**Figure 4A**). These results indicate that a DpR of tumor change of −45% or less could be an independent surrogate marker for better OS and PFS in patients with R/M HNSCC treated with a pembrolizumab-containing regimen. Also by RECIST-based assessment, patients who achieved partial response showed significant better OS and PFS compared with patients who had stable disease or progressive disease (**Figures 4C, D**). However, even among patients who achieved partial response based on RECIST (**Supplementary Figure 3A**), those with deep response of a tumor change of −45% or less had significantly better OS than those with moderate response with a tumor change of −30 to <−45% (median not reached vs. 16.3 months, p=0.013) (**Supplementary Figure 3B**). Among patients who had progressive disease based on RECIST, the median OS for those with tumor change from >20 to ≤80% and >80% were 22.0 and 5.6 months, respectively (hazard rate 0.19, p=0.104) (**Supplementary Figures 3C, D**). Although there was no statistical significance, there was a tendency that patients with progression greater than 80% tumor change rate had a worse prognosis when compared with patients with moderate progression. These data show that DpR may be a more accurate predictor of the clinical outcome than the RECIST-based assessment of response.

**Table 2:** Univariate and multivariate analysis of pembrolizumab-containig regimen (n=66).

	n	OS			PFS		
		HR (95% CI)	p-value		HR (95% CI)	p-value	
			univariate	multivariate		univariate	multivariate
Age							
< 65	20	1	reference		1	reference	
≥ 65	46	1.41 (0.56-3.55)	0.456		1.15 (0.59-2.22)	0.6699	
Sex							
female	11	1	reference		1	reference	
male	55	0.81 (0.30-2.16)	0.676		1.64 (0.63-4.21)	0.3029	
ECOG PS							
≥ 1	47	1	reference		1	reference	
0	19	0.37 (0.16-0.84)	**0.0184**	0.089	0.59 (0.32-1.10)	0.1005	0.542
Primay site							
oral cavity							
no	40	1	reference		1	reference	
yes	26	1.71(0.75-3.78)	0.192		1.12(0.58-2.02)	0.721	
pharynx (hypopharynx, oropharinx)							
no	37	1	reference		1	reference	
yes	29	1.18(0.53-2.61)	0.844		1.01(0.54-1.86)	0.966	
Baseline tumor size							
continuous value	66		0.693	0.745		0.083	0.253
≤ 31	34	1	reference		1	reference	
> 31	32	0.58(0.24-1.29)	0.195		0.56(0.29-1.03)	0.066	
CPS							
< 1 or unknown	11	1	reference		1	reference	
≥ 1	55	0.64 (0.26-1.95)	0.408		1.57(0.66-4.60)	0.322	
< 20 or unknown	32	1	reference		1	reference	
≥ 20	34	1.12 (0.51-2.55)	0.765		1.16 (0.63-2.13)	0.6199	
Locoregional recurrence							
no	49	1	reference		1	reference	
yes	17	2.32 (1.02-5.27)	**0.0442**		2.23 (1.12-4.46)	**0.0222**	0.307
Distant metastasis							
no	15	1	reference		1	reference	
yes	51	0.33 (0.14-0.76)	**0.0095**		0.49 (0.24-1.01)	0.051	
Adverse event (≥Grade 3)							
no	44	1	reference		1	reference	
yes	22	2.08 (0.94-4.60)	0.0698		1.29 (0.42-2.43)	0.4298	
Tumor change rate from base line							
> 0%	30	1	reference		1	reference	
≤ 0% to > -15%	11	0.75 (0.28-1.95)	0.5612		0.40 (0.17-0.91)	**0.0235**	
≤-15% to > -30%	7	0.52 (0.11-2.30)	0.3915		0.47 (0.16-0.87)	0.1418	
≤-30% to > -45%	6	0.35 (0.07-1.55)	0.1679		0.37 (0.12-1.11)	0.377	
≤ -45%	12	NA (0-NA)	**<0.0001**	**0.0001**	0.12 (0.04-0.39)	**0.0003**	**0.0005**

OS, overall survival; PFS, progression free survival; HR, hazard ratio; CI, confidence interval; PS, performance status; CPS, combined positive score; NA, Not Applicable.Statistically significant values are highlighted in bold.

### 3.5 Adverse events

The AEs are summarized in **Table 3**. AEs of any grade occurred in 54 patients (81.8%). Severe AEs were observed in 22 (33.3%) patients. The profile of the immune-related AEs was similar to that reported in the KEYNOTE-048 study (3). As shown in **Table 2**, the occurrence of grade 3 or higher AEs was not associated with the OS or PFS in the univariate and multivariate analyses.

**Table 3:** Adverse events.

pembrolizumab-containing regimen n=66		
	Any grade	Grade ≥3
Any n, (%)	54 (81.8)	22 (33.3)
Blood disorders		
Neutropenia	16 (24.2)	8 (12.1)
Febrile neutropenia	1 (1.5)	1 (1.5)
Anemia	12 (18.2)	8 (12.1)
Thrombocytopenia	7 (10.6)	2 (3)
Endocrine disorders		
Hypothyroidism	4 (6.1)	0
Hypopituitalism	3 (4.5)	0
Gastrointestinal disorder		
Diarrhea	1 (1.5)	0
General disorders		
Decreased appetite	12 (18.2)	4 (6.1)
Fatigue	3 (4.5)	2 (3)
Mucosal inflammation	10 (15.2)	2 (3)
Investigation		
AST increased	2 (3)	0
Metabolism disorders		
hypercalcemia	1 (1.5)	0
hyponatremia	1 (1.5)	0
nervous system disorder		
Peripheral neuropathy	1 (1.5)	0
Respiratory disorder		
Pneumonitis	9 (13.6)	3 (4.5)
Skin disorder		
Rash	5 (7.6)	0

AST, aspartate aminotransfer.

### 3.6 Treatment efficacies of subsequent anticancer therapy

As shown in **Table 1**, 33 (50.0%) patients received subsequent anticancer therapy and 14 (21.2%) patients received best supportive care. The analysis of PFS2 was conducted in the 33 patients who received second-line anticancer therapy. The median PFS2 was 13.9 months (**Supplementary Figure 4A**). Of these, 23 patients who received cetuximab plus paclitaxel also had a median PFS2 of 13.9 months. The overall response rate for all 33 patients and for the 23 patients who received cetuximab plus paclitaxel was 51.5% and 65.2%, respectively (**Supplementary Table 1**). The transition rate to second-line anticancer therapy represents the percentage of patients who receive second-line anticancer therapy after first-line therapy discontinuation. The transition rate to second-line anticancer therapy was 70.2% in this study (**Supplementary Table 1**). PFS2, response rate, and the transition rate to second-line anticancer therapy were evaluated by the DpR to first-line pembrolizumab-containing regimens. The DpR to the first-line therapy was not associated with PFS2 (**Supplementary Figure 4B**) or response to second-line therapy, but tended to be associated with transition rate to second-line anticancer therapy (**Supplementary Table 1**). Patients with tumor shrinkage in first-line therapy tended to have a higher rate of transition to second-line anticancer therapy (81.4% vs. 55.0%, p=0.05). The 33 patients who received second-line anticancer therapy had significantly better OS than 14 patients who received best supportive care (hazard rate 0.23, p=0.0007) (**Supplementary Figure 4C**). Patients who achieve a response to first-line therapy have a higher rate of transition to second-line therapy, which may also contribute to longer survival.

## 4 Discussion

The predictive value of DpR for HNSCC has not yet been reported. In the present study, we focused on investigating the effects of DpR on the survival of patients with R/M HNSCC receiving pembrolizumab-containing regimens.

The pembrolizumab-containing regimen had a favorable survival benefit with a 1 year-OS rate of 69.4% and a 1 year-PFS rate of 24.4% (**Figure 1**). These results are comparable to previously reported real-world data in Japanese patients (11, 12). Evaluation of the DpR using a waterfall plot showed that pembrolizumab with chemotherapy resulted in a better response compared with pembrolizumab alone (**Figure 2**). The median time to response was 2.0 months. The median time to best response for the measurement of DpR was 2.8 months. The time to response was almost equal to that of the KEYNOTE048 study (5). The DpR to pembrolizumab-containing regimens was correlated with both OS and PFS (**Figure 3**). Even if a separate analysis was performed for pembrolizumab with chemotherapy and pembrolizumab alone, significant correlations were still observed between DpR and survival (**Supplementary Figure 2**). In the multivariate analysis, a deep response with a change of −45% or greater was an independent predictive factor for OS and PFS (**Table 2**). Although the association between response based on the RECIST criteria and survival has been reported (12), this study is the first to report an association between DpR and survival following pembrolizumab-containing regimens in patients with R/M HNSCC. In addition, our results show that DpR may be able to predict survival outcomes more precisely than responses evaluated based on RECIST (**Supplementary Figure 3**).

As for other ICI regimens, the efficacy of nivolumab has been associated with pre-treatment tumor size in patients with previously treated R/M HNSCC (13). In that study, tumor size was assessed including all measurable lesions (not based on RECIST) (13). In some cases, use of the RECIST criteria is not sufficient to accurately determine the tumor volume, owing to the limited number of target lesions in a single organ and exclusion of small lymph nodes with a diameter of <10 mm from the criteria (9). That may be why baseline tumor size was not associated with OS or PFS in this study (**Table 2**). DpR may compensate for the insufficiency of RECIST-based evaluation of baseline tumor size in predicting survival outcome. Other reports have suggested that ICIs can exert long-lasting effects when the tumor volume in patients with lung cancer and melanoma is small (14, 15). Our results are consistent with those of previous studies. A deep response to first-line therapy reduces the tumor volume, thus increasing the efficacy of ICIs.

In this study, the CPS was not associated with OS or PFS (**Table 2**). We assume that this is due to the appropriate selection of treatment regimens based on the CPS. All CPS-negative patients received pembrolizumab with chemotherapy, not pembrolizumab alone. Locoregional recurrence was a poor predictive factor in the univariate analyses of OS and PFS (**Table 2**). A subgroup analysis of OS in the KEYNOTE-048 study also demonstrated the favorable effect of pembrolizumab in patients with metastatic disease rather than in those with locoregional recurrence (4). Locoregional recurrence can induce the occurrence of symptoms that impair the quality of life and PS and sometimes leads to fatal events, such as bleeding and choking (16). For patients with locoregional recurrence, alternative regimens such as cetuximab-containing regimens may be suitable.

The median PFS2 in this study was 13.9 months, which was better than the reported PFS2 for patients with CPS ≥ 20 in the KEYNOTE-048 study (4). The most common subsequent regimen was cetuximab plus paclitaxel in this study. In contrast, cetuximab was used in only about 20% of patients in the KEYNOTE-048 study (4). This difference may have influenced the results. Interestingly, the DpR in the first-line therapy was not associated with PFS2 (**Supplementary Figure 4B**), but tended to be associated with higher transition rate to second-line anticancer therapy (**Supplementary Table 1**). Future studies with larger numbers of patients are needed to confirm the association.

This study has several limitations. First, this was a retrospective study conducted in a single institution with a small number of patients. In the subgroup analysis, the number of patients was even smaller, which have limited the statistical power of the analysis. Second, major analyses only examined the effects of pembrolizumab regimens that included both pembrolizumab with chemotherapy and pembrolizumab alone. Third, there was some bias in the patients’ characteristics in this study. Patients in this study were more likely to have distant metastases and older when compared to previously reported real-world data (10, 12). The possibility that such bias may have affected treatment efficacy must be considered. There was also a biased selection of the platinum agents. Most of patients treated with pembrolizumab with chemotherapy received carboplatin-based chemotherapy. Generally, carboplatin represents a better tolerated alternative to cisplatin. Although the superior survival with cisplatin over carboplatin was reported in the EXTREME study, there have been no investigations on the choice of the platinum agents in combination with pembrolizumab (17). In the KEYNOTE-048 study, about 60% of patients received carboplatin-based chemotherapy (3). For those reasons, carboplatin was predominantly chosen in our institute.

In conclusion, the DpR induced by first-line pembrolizumab-containing regimens may be a predictive factor for OS and PFS in patients with R/M HNSCC. Hence, further research is warranted to confirm whether the DpR in pembrolizumab treatment is important in deciding the total treatment strategy for R/M HNSCC.

## Data availability statement

The original contributions presented in the study are included in the article/**Supplementary Material**. Further inquiries can be directed to the corresponding author.

## Ethics statement

This study was approved by the Ethics Committee of the Faculty of Medicine of the Tohoku University School of Medicine. This study was conducted in accordance with the local legislation and institutional requirements. Written informed consent for participation was not required from the participants or the participants’ legal guardians/next of kin because this was a retrospective study. Patient consent for this study was obtained using the opt-out method. The ethics committee has approved this.

## Author contributions

KSai contributed to the conception and design of the study. All authors treated patients and collected clinical data. KSai, HI, and KO conducted statistical analyses. KSai drafted the original manuscript. CI reviewed the study. All authors contributed to the article and approved the submitted version.
