# Supplementary figures and images for: Depth of response may predict clinical outcome in patients with recurrent/metastatic head and neck cancer treated with pembrolizumab-containing regimens
Source: Front Oncol. 2023 Aug 16;13:1230731. doi: 10.3389/fonc.2023.1230731 (PMC10469278; doi:10.3389/fonc.2023.1230731)

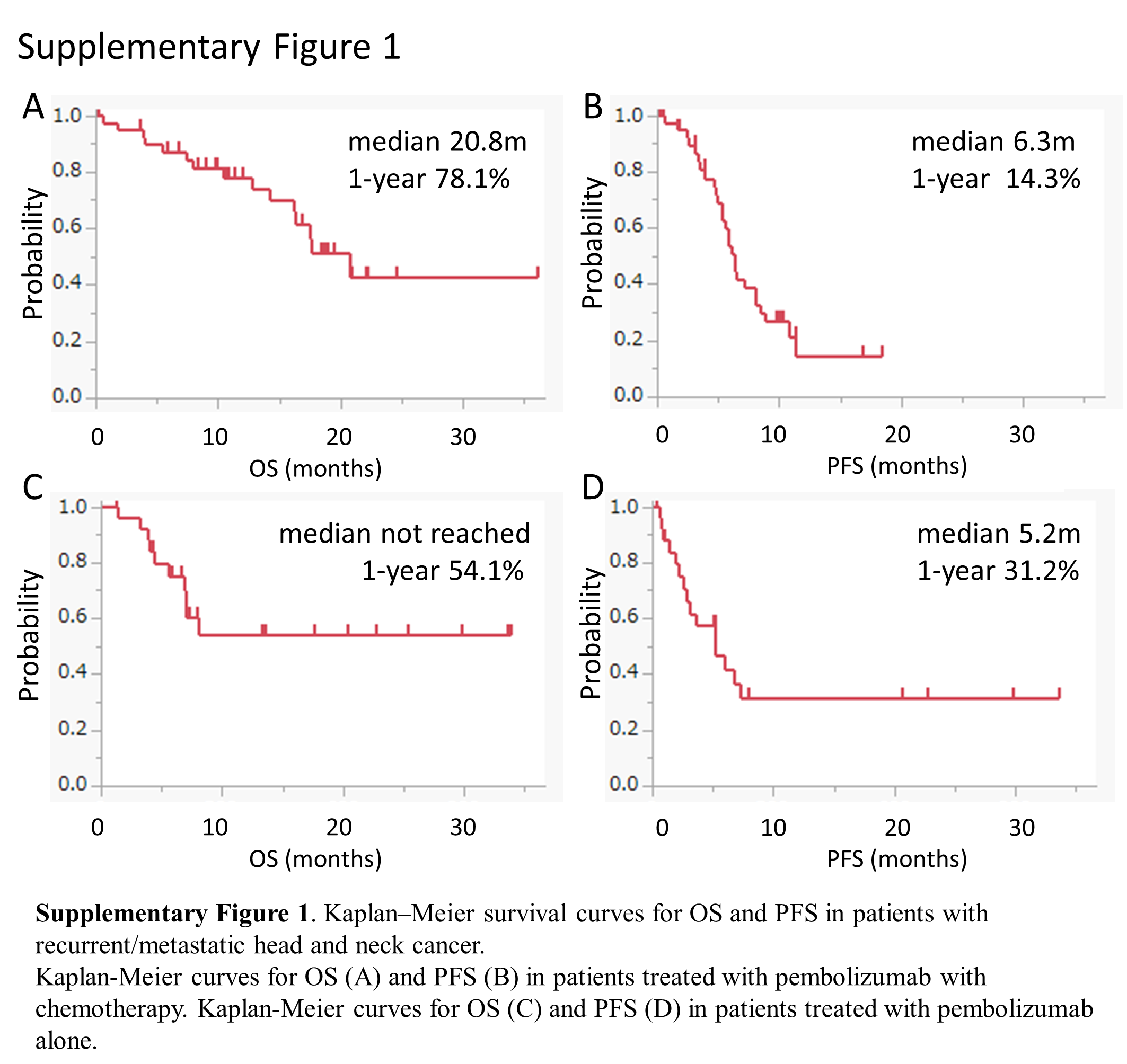

Supplement: Supplementary file 1 [file Image_1.tif]

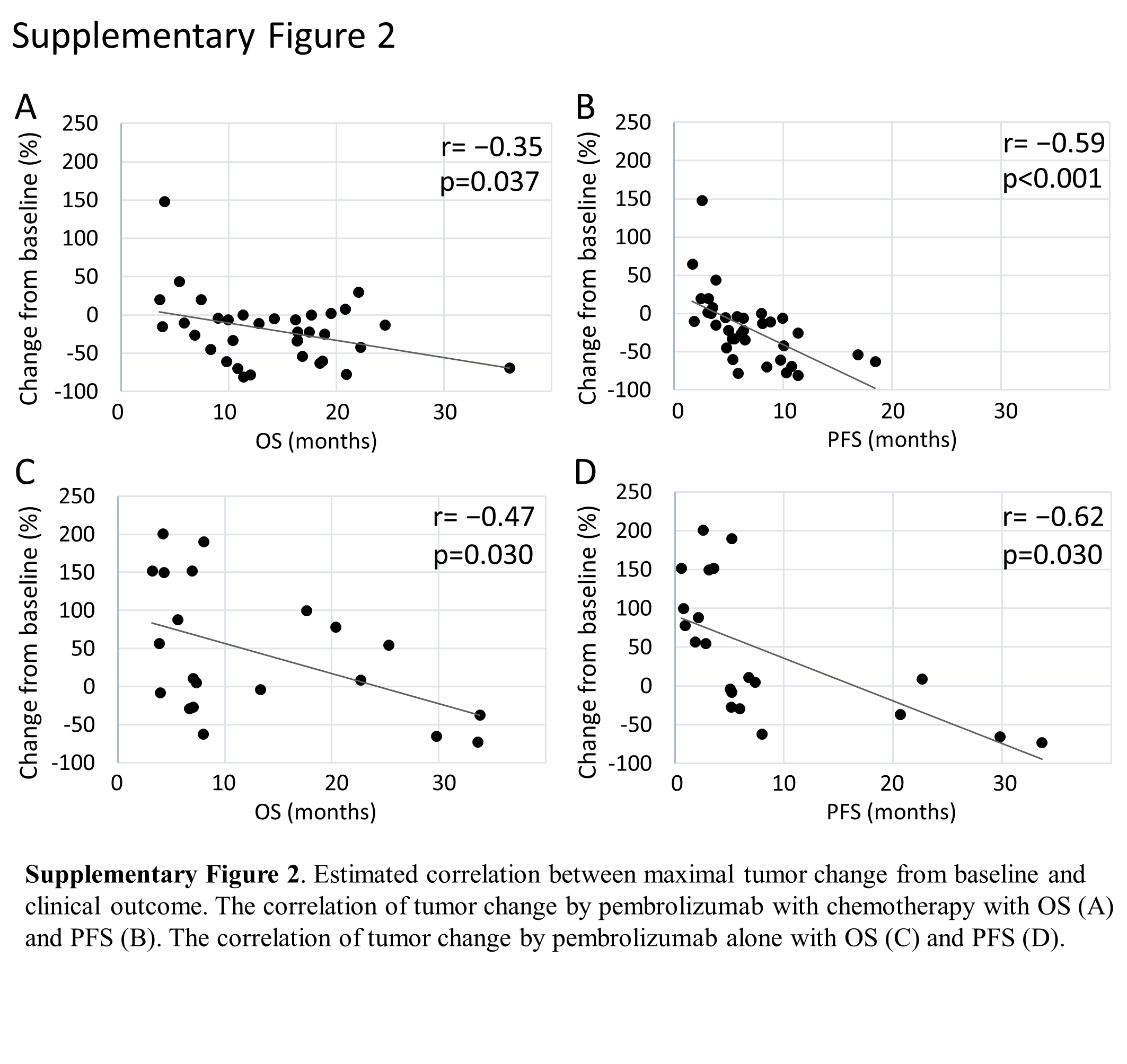

Supplement: Supplementary file 2 [file Image_2.tif]

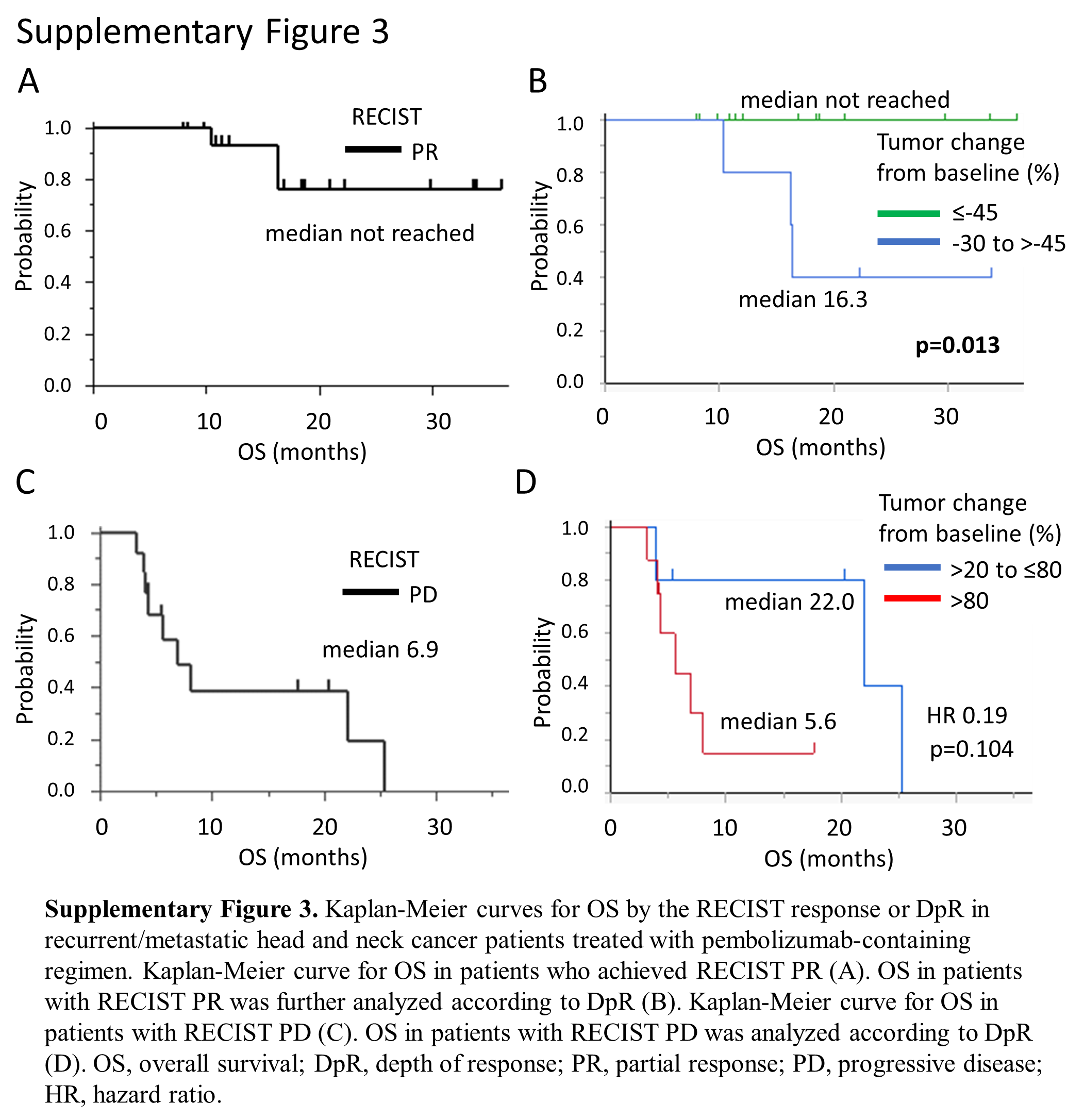

Supplement: Supplementary file 3 [file Image_3.tif]

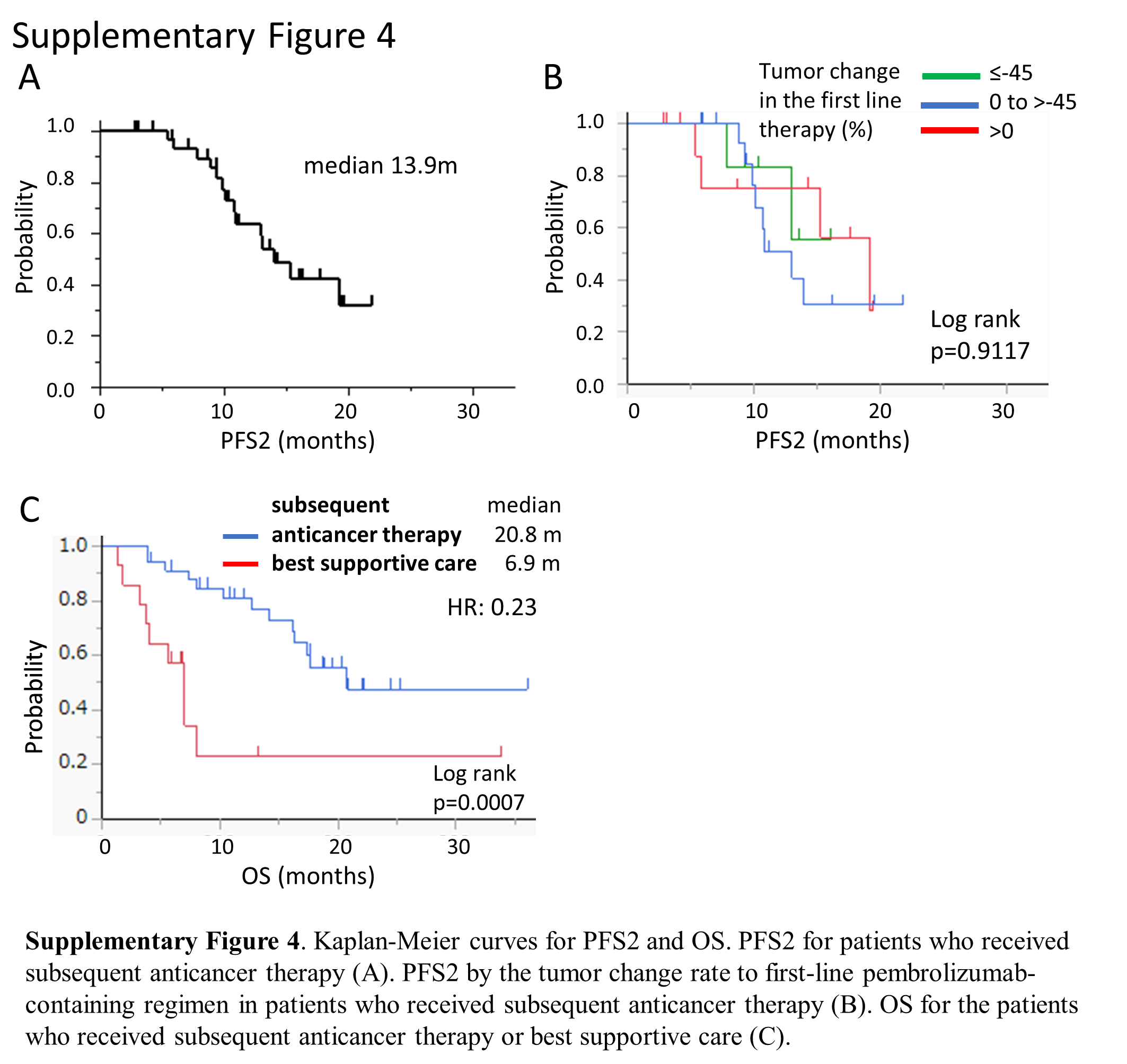

Supplement: Supplementary file 4 [file Image_4.tif]
