# Supplementary material for: Depth of response may predict clinical outcome in patients with recurrent/metastatic head and neck cancer treated with pembrolizumab-containing regimens
Source: Front Oncol. 2023 Aug 16;13:1230731. doi: 10.3389/fonc.2023.1230731 (PMC10469278; doi:10.3389/fonc.2023.1230731)
Supplement: Supplementary file 5 [file Table_1.pdf]

Supplementary Table 1

subsequent therapy		tumor change in first-line therapy (%)		Fisher's exact test (p-value)
		0>	≤0	
total (n)	47	20	27	
Anticancer therapy (n)	33	11	22	
PR (n)	17	6	11	
SD (n)	5	2	3	
PD (n)	5	1	4	
NE (n)	6	2	4	
ORR (%)	51.5	54.5	50.0	0.549
Best supportive care (n)	14	9	5	
Transition rate to second-line anticancer therapy (%)	70.2	55.0	81.4	0.050

PR, partial response; PD, progressive disease; NE, not evaluated; ORR, overall response rate. The transition rate to second-line anticancer therapy was calculated by dividing the number of patients who received second-line anticancer therapy by the number of patients who received subsequent therapy including best supportive care.
